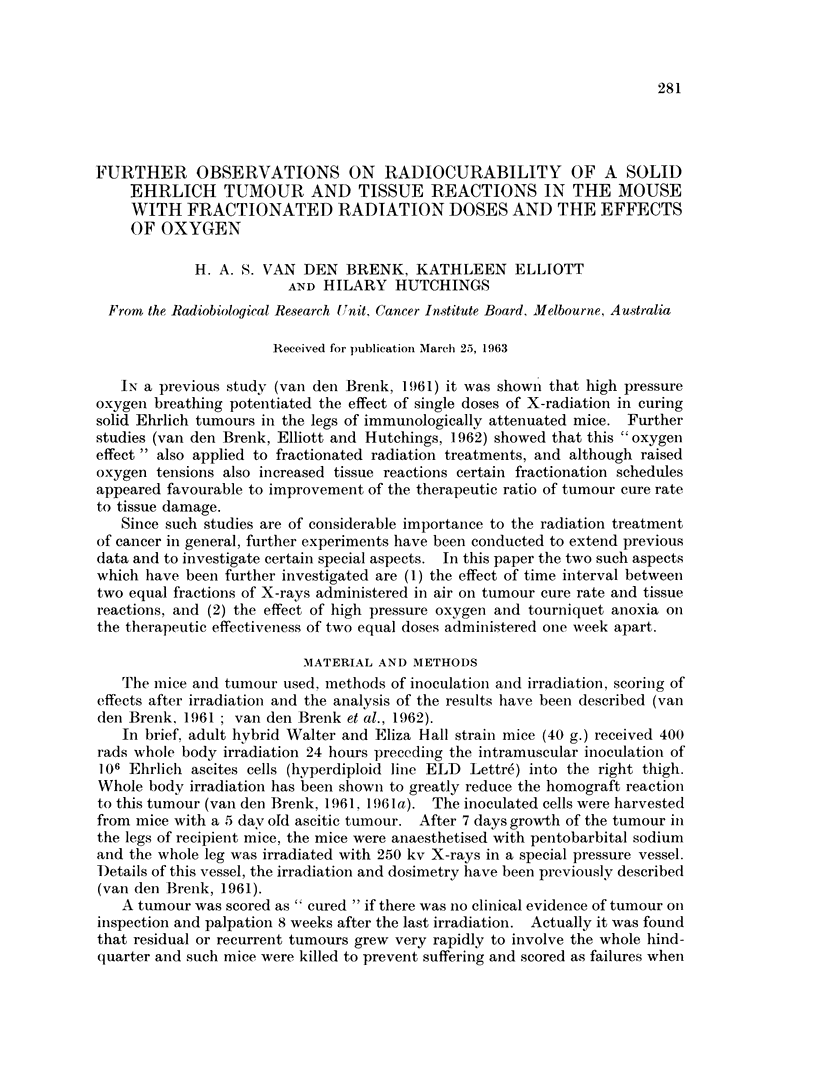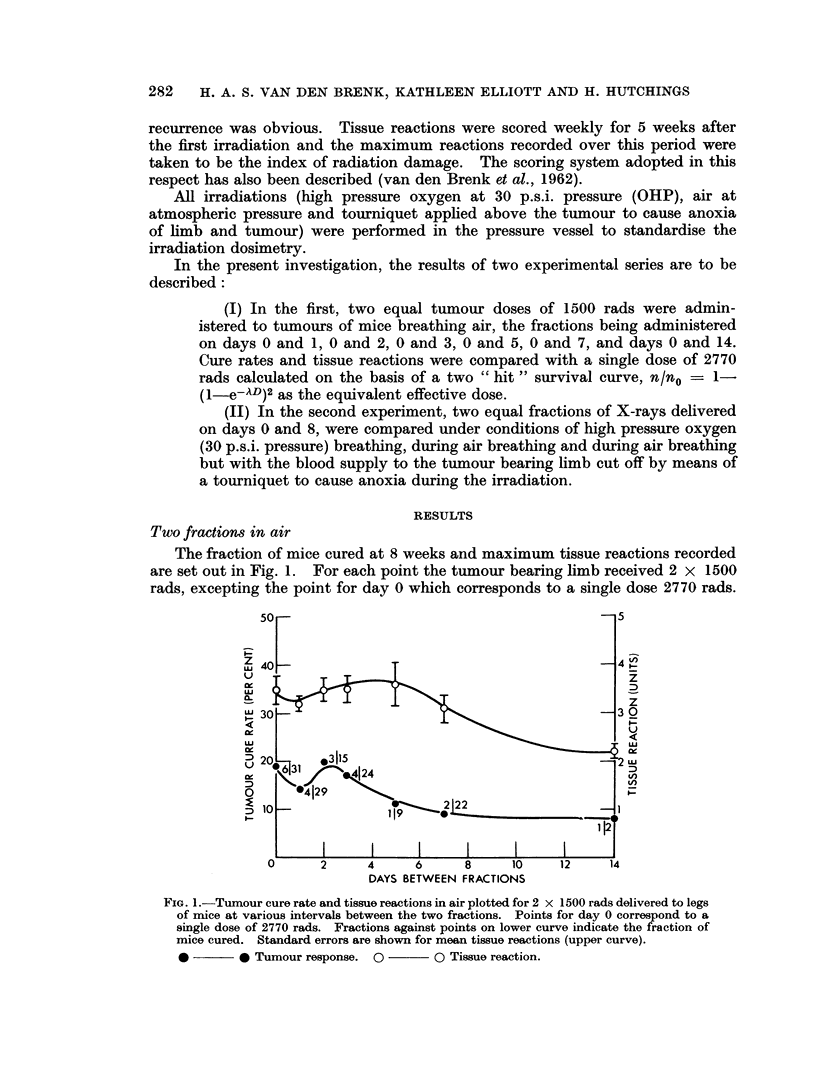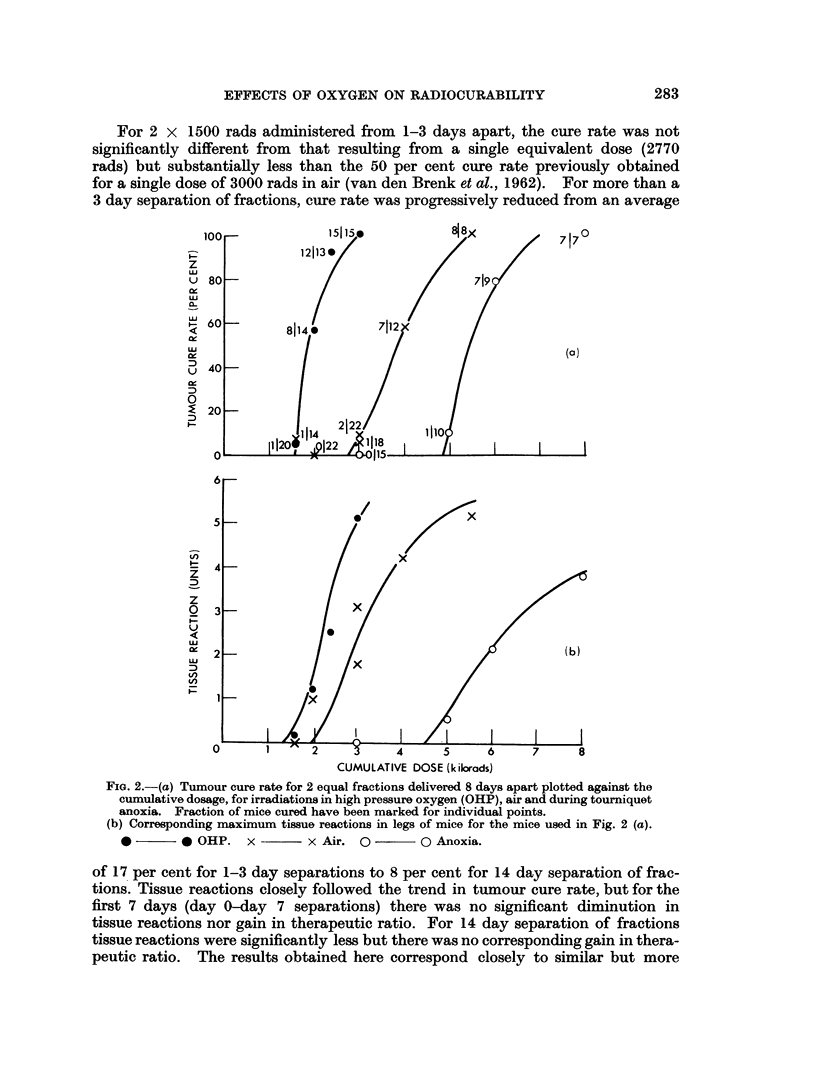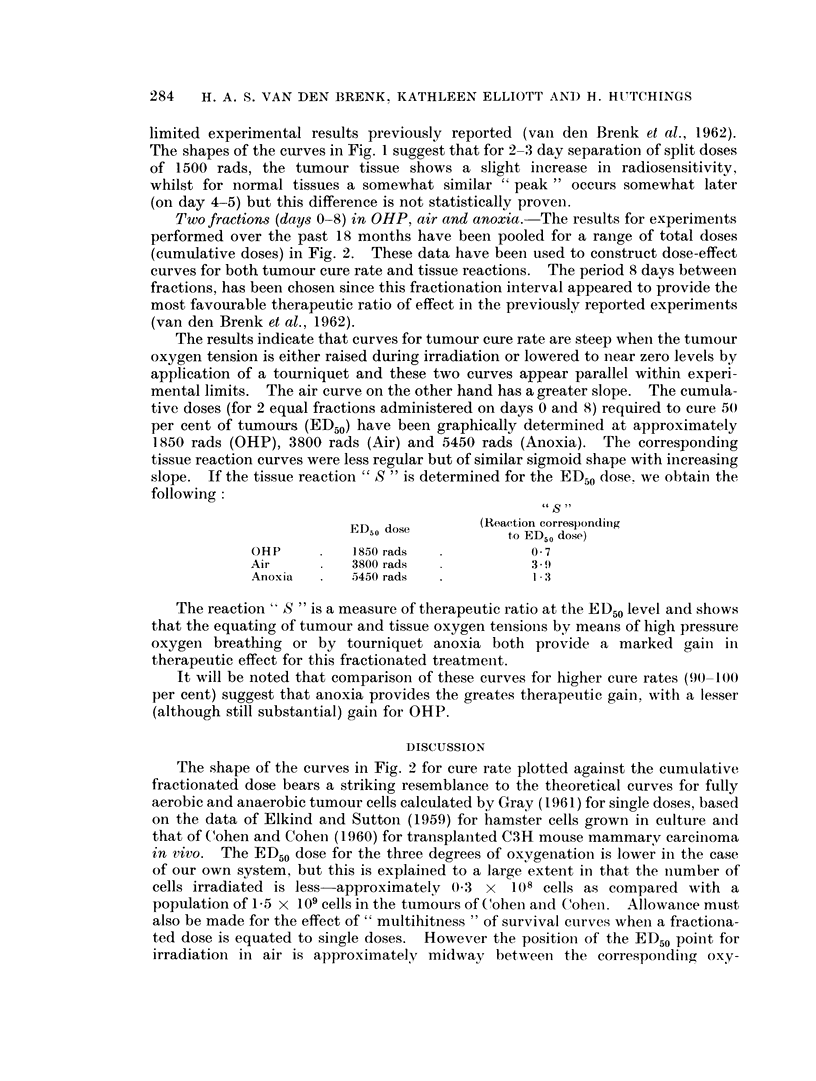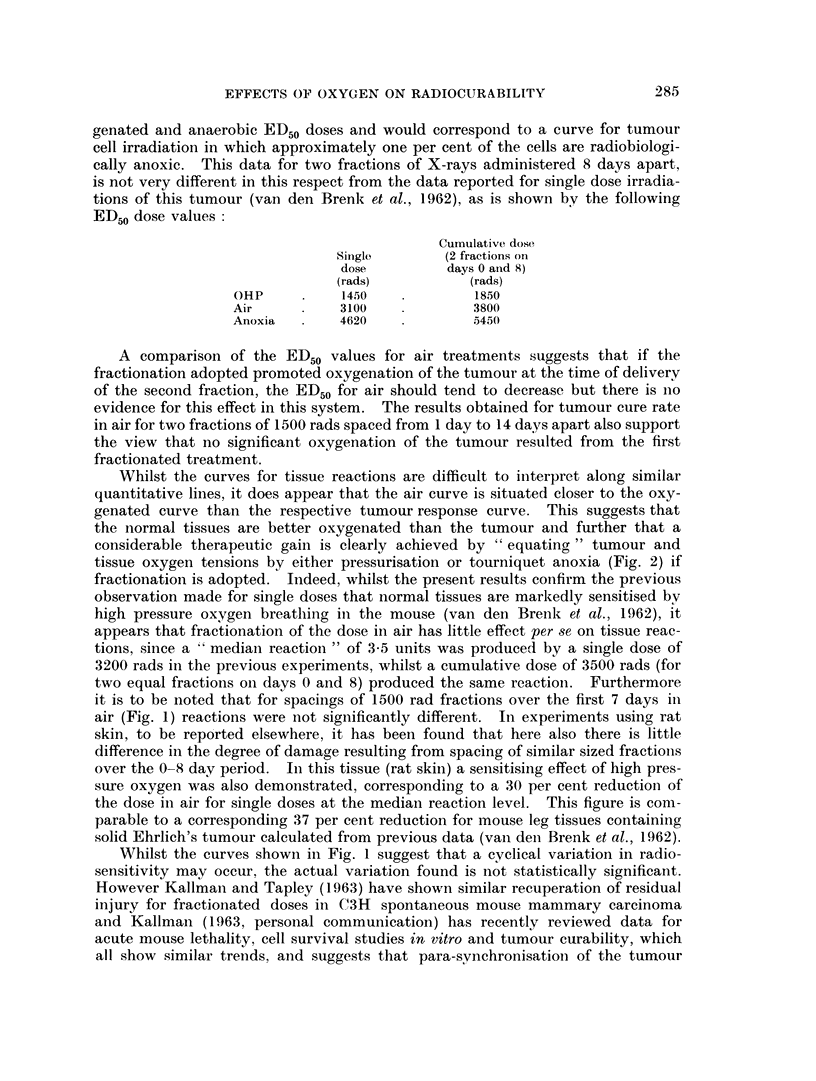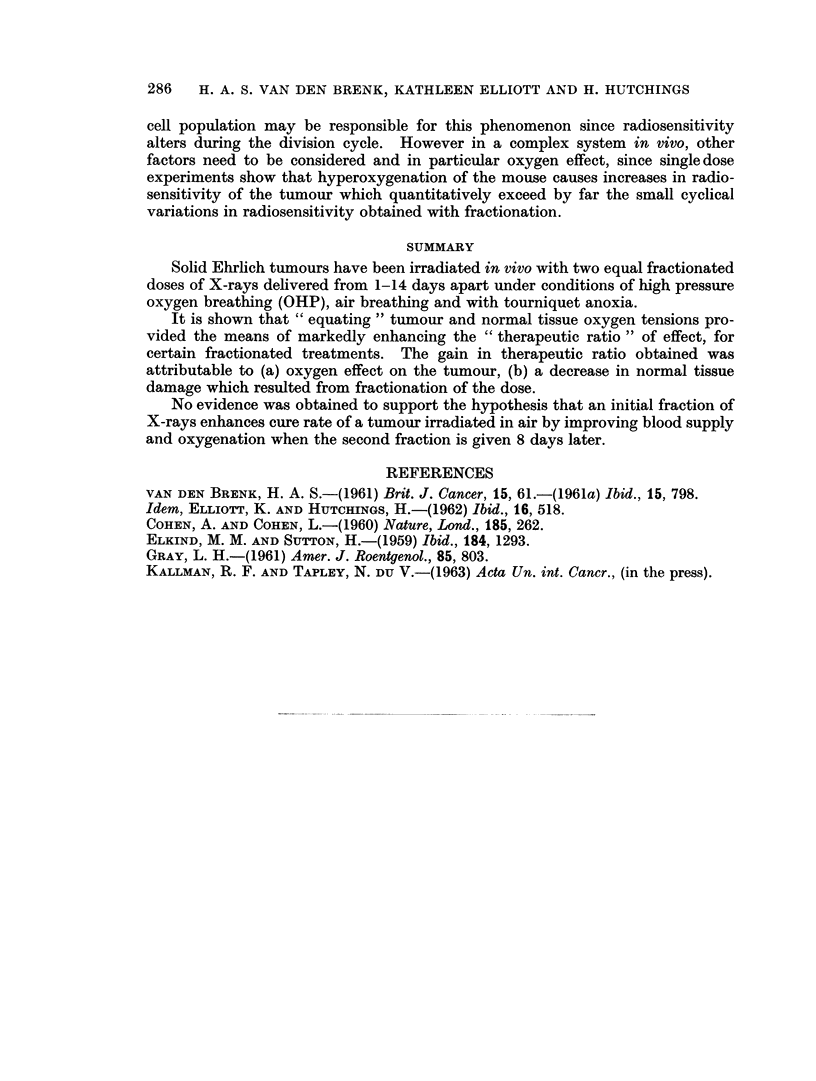# Further Observations on Radiocurability of a Solid Ehrlich Tumour and Tissue Reactions in the Mouse with Fractionated Radiation Doses and the Effects of Oxygen

**DOI:** 10.1038/bjc.1963.40

**Published:** 1963-06

**Authors:** H. A. S. van den Brenk, Kathleen Elliott, Hilary Hutchings


### FURTHER OBSERVATIONS ON RADIOCURABILITY OF A SOLID EHRLICH TUMOUR AND TISSUE REACTIONS IN THE MOUSE WITH FRACTIONATED RADIATION DOSES AND THE EFFECTS OF OXYGEN

H. A. S. VAN DEN BRENK, KATHLEEN ELLIOTT AND HILARY HUTCHINGS

*From the Radiobiological Research Unit, Cancer Institute Board, Melbourne, Australia*

Received for publication March 25, 1963

IN a previous study (van den Brenk, 1961) it was shown that high pressure oxygen breathing potentiated the effect of single doses of X-radiation in curing solid Ehrlich tumours in the legs of immunologically attenuated mice. Further studies (van den Brenk, Elliott and Hutchings, 1962) showed that this "oxygen effect" also applied to fractionated radiation treatments, and although raised oxygen tensions also increased tissue reactions certain fractionation schedules appeared favourable to improvement of the therapeutic ratio of tumour cure rate to tissue damage.

Since such studies are of considerable importance to the radiation treatment of cancer in general, further experiments have been conducted to extend previous data and to investigate certain special aspects. In this paper the two such aspects which have been further investigated are (1) the effect of time interval between two equal fractions of X-rays administered in air on tumour cure rate and tissue reactions, and (2) the effect of high pressure oxygen and tourniquet anoxia on the therapeutic effectiveness of two equal doses administered one week apart.

### MATERIAL AND METHODS

The mice and tumour used, methods of inoculation and irradiation, scoring of effects after irradiation and the analysis of the results have been described (van den Brenk, 1961 ; van den Brenk *et al.*, 1962).

In brief, adult hybrid Walter and Eliza Hall strain mice (40 g.) received 400 rads whole body irradiation 24 hours preceding the intramuscular inoculation of $10^6$ Ehrlich ascites cells (hyperdiploid line ELD Lettré) into the right thigh. Whole body irradiation has been shown to greatly reduce the homograft reaction to this tumour (van den Brenk, 1961, 1961*a*). The inoculated cells were harvested from mice with a 5 day old ascitic tumour. After 7 days growth of the tumour in the legs of recipient mice, the mice were anaesthetised with pentobarbital sodium and the whole leg was irradiated with 250 kv X-rays in a special pressure vessel. Details of this vessel, the irradiation and dosimetry have been previously described (van den Brenk, 1961).

A tumour was scored as "cured" if there was no clinical evidence of tumour on inspection and palpation 8 weeks after the last irradiation. Actually it was found that residual or recurrent tumours grew very rapidly to involve the whole hind-quarter and such mice were killed to prevent suffering and scored as failures when recurrence was obvious. Tissue reactions were scored weekly for 5 weeks after the first irradiation and the maximum reactions recorded over this period were taken to be the index of radiation damage. The scoring system adopted in this respect has also been described (van den Brenk *et al.*, 1962).

All irradiations (high pressure oxygen at 30 p.s.i. pressure (OHP), air at atmospheric pressure and tourniquet applied above the tumour to cause anoxia of limb and tumour) were performed in the pressure vessel to standardise the irradiation dosimetry.

In the present investigation, the results of two experimental series are to be described :

(I) In the first, two equal tumour doses of 1500 rads were administered to tumours of mice breathing air, the fractions being administered on days 0 and 1, 0 and 2, 0 and 3, 0 and 5, 0 and 7, and days 0 and 14. Cure rates and tissue reactions were compared with a single dose of 2770 rads calculated on the basis of a two " hit " survival curve, $n/n_0 = 1 - (1 - e^{-\lambda D})^2$ as the equivalent effective dose.

(II) In the second experiment, two equal fractions of X-rays delivered on days 0 and 8, were compared under conditions of high pressure oxygen (30 p.s.i. pressure) breathing, during air breathing and during air breathing but with the blood supply to the tumour bearing limb cut off by means of a tourniquet to cause anoxia during the irradiation.

### RESULTS

### *Two fractions in air*

The fraction of mice cured at 8 weeks and maximum tissue reactions recorded are set out in Fig. 1. For each point the tumour bearing limb received 2 × 1500 rads, excepting the point for day 0 which corresponds to a single dose 2770 rads.

FIG. 1.—Tumour cure rate and tissue reactions in air plotted for 2 × 1500 rads delivered to legs of mice at various intervals between the two fractions. Points for day 0 correspond to a single dose of 2770 rads. Fractions against points on lower curve indicate the fraction of mice cured. Standard errors are shown for mean tissue reactions (upper curve).

● ——— ● Tumour response. ○ ——— ○ Tissue reaction.

For 2 × 1500 rads administered from 1–3 days apart, the cure rate was not significantly different from that resulting from a single equivalent dose (2770 rads) but substantially less than the 50 per cent cure rate previously obtained for a single dose of 3000 rads in air (van den Brenk *et al.*, 1962). For more than a 3 day separation of fractions, cure rate was progressively reduced from an average

FIG. 2.—(*a*) Tumour cure rate for 2 equal fractions delivered 8 days apart plotted against the cumulative dosage, for irradiations in high pressure oxygen (OHP), air and during tourniquet anoxia. Fraction of mice cured have been marked for individual points.

(b) Corresponding maximum tissue reactions in legs of mice for the mice used in Fig. 2 (*a*).

● ——— ● OHP. × ——— × Air. ○ ——— ○ Anoxia.

of 17 per cent for 1–3 day separations to 8 per cent for 14 day separation of fractions. Tissue reactions closely followed the trend in tumour cure rate, but for the first 7 days (day 0–day 7 separations) there was no significant diminution in tissue reactions nor gain in therapeutic ratio. For 14 day separation of fractions tissue reactions were significantly less but there was no corresponding gain in therapeutic ratio. The results obtained here correspond closely to similar but more limited experimental results previously reported (van den Brenk *et al.*, 1962). The shapes of the curves in Fig. 1 suggest that for 2–3 day separation of split doses of 1500 rads, the tumour tissue shows a slight increase in radiosensitivity, whilst for normal tissues a somewhat similar " peak " occurs somewhat later (on day 4–5) but this difference is not statistically proven.

*Two fractions (days 0–8) in OHP, air and anoxia.*—The results for experiments performed over the past 18 months have been pooled for a range of total doses (cumulative doses) in Fig. 2. These data have been used to construct dose-effect curves for both tumour cure rate and tissue reactions. The period 8 days between fractions, has been chosen since this fractionation interval appeared to provide the most favourable therapeutic ratio of effect in the previously reported experiments (van den Brenk *et al.*, 1962).

The results indicate that curves for tumour cure rate are steep when the tumour oxygen tension is either raised during irradiation or lowered to near zero levels by application of a tourniquet and these two curves appear parallel within experimental limits. The air curve on the other hand has a greater slope. The cumulative doses (for 2 equal fractions administered on days 0 and 8) required to cure 50 per cent of tumours ($ED_{50}$) have been graphically determined at approximately 1850 rads (OHP), 3800 rads (Air) and 5450 rads (Anoxia). The corresponding tissue reaction curves were less regular but of similar sigmoid shape with increasing slope. If the tissue reaction " *S* " is determined for the $ED_{50}$ dose, we obtain the following :

| | $ED_{50}$ dose | " *S* " (Reaction corresponding to $ED_{50}$ dose) |
|---|---|---|
| OHP | 1850 rads | 0·7 |
| Air | 3800 rads | 3·9 |
| Anoxia | 5450 rads | 1·3 |

The reaction " *S* " is a measure of therapeutic ratio at the $ED_{50}$ level and shows that the equating of tumour and tissue oxygen tensions by means of high pressure oxygen breathing or by tourniquet anoxia both provide a marked gain in therapeutic effect for this fractionated treatment.

It will be noted that comparison of these curves for higher cure rates (90–100 per cent) suggest that anoxia provides the greates therapeutic gain, with a lesser (although still substantial) gain for OHP.

### DISCUSSION

The shape of the curves in Fig. 2 for cure rate plotted against the cumulative fractionated dose bears a striking resemblance to the theoretical curves for fully aerobic and anaerobic tumour cells calculated by Gray (1961) for single doses, based on the data of Elkind and Sutton (1959) for hamster cells grown in culture and that of Cohen and Cohen (1960) for transplanted C3H mouse mammary carcinoma *in vivo*. The $ED_{50}$ dose for the three degrees of oxygenation is lower in the case of our own system, but this is explained to a large extent in that the number of cells irradiated is less—approximately $0 \cdot 3 \times 10^8$ cells as compared with a population of $1 \cdot 5 \times 10^9$ cells in the tumours of Cohen and Cohen. Allowance must also be made for the effect of " multihitness " of survival curves when a fractionated dose is equated to single doses. However the position of the $ED_{50}$ point for irradiation in air is approximately midway between the corresponding oxygenated and anaerobic $ED_{50}$ doses and would correspond to a curve for tumour cell irradiation in which approximately one per cent of the cells are radiobiologically anoxic. This data for two fractions of X-rays administered 8 days apart, is not very different in this respect from the data reported for single dose irradiations of this tumour (van den Brenk *et al.*, 1962), as is shown by the following $ED_{50}$ dose values :

| | Single dose (rads) | Cumulative dose (2 fractions on days 0 and 8) (rads) |
|---|---|---|
| OHP | 1450 | 1850 |
| Air | 3100 | 3800 |
| Anoxia | 4620 | 5450 |

A comparison of the $ED_{50}$ values for air treatments suggests that if the fractionation adopted promoted oxygenation of the tumour at the time of delivery of the second fraction, the $ED_{50}$ for air should tend to decrease but there is no evidence for this effect in this system. The results obtained for tumour cure rate in air for two fractions of 1500 rads spaced from 1 day to 14 days apart also support the view that no significant oxygenation of the tumour resulted from the first fractionated treatment.

Whilst the curves for tissue reactions are difficult to interpret along similar quantitative lines, it does appear that the air curve is situated closer to the oxygenated curve than the respective tumour response curve. This suggests that the normal tissues are better oxygenated than the tumour and further that a considerable therapeutic gain is clearly achieved by " equating " tumour and tissue oxygen tensions by either pressurisation or tourniquet anoxia (Fig. 2) if fractionation is adopted. Indeed, whilst the present results confirm the previous observation made for single doses that normal tissues are markedly sensitised by high pressure oxygen breathing in the mouse (van den Brenk *et al.*, 1962), it appears that fractionation of the dose in air has little effect *per se* on tissue reactions, since a " median reaction " of 3·5 units was produced by a single dose of 3200 rads in the previous experiments, whilst a cumulative dose of 3500 rads (for two equal fractions on days 0 and 8) produced the same reaction. Furthermore it is to be noted that for spacings of 1500 rad fractions over the first 7 days in air (Fig. 1) reactions were not significantly different. In experiments using rat skin, to be reported elsewhere, it has been found that here also there is little difference in the degree of damage resulting from spacing of similar sized fractions over the 0–8 day period. In this tissue (rat skin) a sensitising effect of high pressure oxygen was also demonstrated, corresponding to a 30 per cent reduction of the dose in air for single doses at the median reaction level. This figure is comparable to a corresponding 37 per cent reduction for mouse leg tissues containing solid Ehrlich's tumour calculated from previous data (van den Brenk *et al.*, 1962).

Whilst the curves shown in Fig. 1 suggest that a cyclical variation in radiosensitivity may occur, the actual variation found is not statistically significant. However Kallman and Tapley (1963) have shown similar recuperation of residual injury for fractionated doses in C3H spontaneous mouse mammary carcinoma and Kallman (1963, personal communication) has recently reviewed data for acute mouse lethality, cell survival studies *in vitro* and tumour curability, which all show similar trends, and suggests that para-synchronisation of the tumour cell population may be responsible for this phenomenon since radiosensitivity alters during the division cycle. However in a complex system *in vivo*, other factors need to be considered and in particular oxygen effect, since single dose experiments show that hyperoxygenation of the mouse causes increases in radiosensitivity of the tumour which quantitatively exceed by far the small cyclical variations in radiosensitivity obtained with fractionation.

### SUMMARY

Solid Ehrlich tumours have been irradiated *in vivo* with two equal fractionated doses of X-rays delivered from 1–14 days apart under conditions of high pressure oxygen breathing (OHP), air breathing and with tourniquet anoxia.

It is shown that " equating " tumour and normal tissue oxygen tensions provided the means of markedly enhancing the " therapeutic ratio " of effect, for certain fractionated treatments. The gain in therapeutic ratio obtained was attributable to (a) oxygen effect on the tumour, (b) a decrease in normal tissue damage which resulted from fractionation of the dose.

No evidence was obtained to support the hypothesis that an initial fraction of X-rays enhances cure rate of a tumour irradiated in air by improving blood supply and oxygenation when the second fraction is given 8 days later.

### REFERENCES

VAN DEN BRENK, H. A. S.—(1961) *Brit. J. Cancer*, **15**, 61.—(1961*a*) *Ibid.*, **15**, 798.

*Idem*, ELLIOTT, K. AND HUTCHINGS, H.—(1962) *Ibid.*, **16**, 518.

COHEN, A. AND COHEN, L.—(1960) *Nature, Lond.*, **185**, 262.

ELKIND, M. M. AND SUTTON, H.—(1959) *Ibid.*, **184**, 1293.

GRAY, L. H.—(1961) *Amer. J. Roentgenol.*, **85**, 803.

KALLMAN, R. F. AND TAPLEY, N. DU V.—(1963) *Acta Un. int. Cancr.*, (in the press).